# What Do We Know About Cyclooxygenases Expression in the Lower Urinary Tract of Dogs and Cats? A Systematic Review

**DOI:** 10.1111/jvp.70090

**Published:** 2026-07-01

**Authors:** Reiner Silveira de Moraes, Luíz Guilherme Dércore Benevenuto, Ana Giulia Gabriel da Rocha Cesario, Julia Franco Ferreira, Suellen Rodrigues Maia, Doughlas Regalin, Dirceu Guilherme de Souza Ramos, Alessandra Melchert, Priscylla Tatiana Chalfun Guimarães‐Okamoto

**Affiliations:** ^1^ Department of Veterinary Clinics, School of Veterinary Medicine and Animal Science São Paulo State University (UNESP) Botucatu Brazil; ^2^ Department of Veterinary Surgery and Anesthesiology, School of Veterinary Medicine and Animal Science São Paulo State University (UNESP) Botucatu Brazil; ^3^ Department of Veterinary Medicine Federal Institute of South Minas (IFSULDEMINAS) Muzambinho Brazil; ^4^ School of Veterinary Medicine and Animal Bioscience Federal University of Jataí (UFJ) Jataí Brazil

**Keywords:** bladder, cats, COX‐1, COX‐2, dogs, inflammation, urethra

## Abstract

Cyclooxygenases (COX‐1 and COX‐2) are key enzymes in the biosynthesis of prostanoids, which mediate physiological functions, inflammation, and the process of carcinogenesis. In dogs and cats, lower urinary tract diseases are prevalent and often complex, requiring targeted therapeutic interventions. Although the role of cyclooxygenases is well established in humans, their expression profile in dogs and cats remains underexplored. This systematic review aimed to consolidate the available knowledge regarding the expression of COX‐1 and COX‐2 in the lower urinary tract tissues of dogs and cats, highlighting pathological implications and gaps in the literature. A total of 28 original studies were included in this review, which employed methodologies such as immunohistochemistry (IHC), Western blotting (WB), in situ hybridization (ISH), RNA sequencing, and ELISA. The urinary bladder was the most frequently analyzed tissue, followed by the urethra. COX‐2 expression was predominantly elevated in tissues with inflammation or neoplasia, whereas COX‐1 exhibited a constitutive expression pattern in normal tissues. In cats with feline idiopathic cystitis (FIC), COX‐2 expression was significantly elevated in the urethral mucosa. In cases of transitional cell carcinoma (TCC), COX‐2 expression varied substantially. The consistent expression of COX‐1 in both normal and altered tissues suggested a homeostatic role. Despite the potential of COX‐2 as a therapeutic target and prognostic marker, methodological heterogeneity, small sample sizes, and the lack of standardized criteria limit the comparability of findings. This review underscores the need for well‐designed studies and standardized approaches to better understand the role of COX enzymes in the uropathology of small animals.

## 1 Introduction

Lower urinary tract disorders represent a common group of conditions encountered in small animal clinical practice, affecting both dogs and cats. Regardless of etiology, these conditions often share similar clinical manifestations, such as dysuria, hematuria, pollakiuria, and inappropriate urination (Lee and Drobatz 2003). In felines, feline lower urinary tract disease (FLUTD) accounts for up to 13% of visits to primary veterinary care services (Lekcharoensuk et al. 2001; Lee and Drobatz 2003). Among the possible causes, feline idiopathic cystitis (FIC) is the most prevalent, accounting for approximately 55% to 69% of cases after the exclusion of specific etiologies such as bacterial infections, urolithiasis, and neoplasia (Sævik et al. 2011).

The FIC is considered an intrinsic idiopathic condition of domestic cats, the etiopathogenesis of which remains incompletely understood. Evidence suggests that structural and molecular alterations in the urothelium, such as changes in the expression of barrier proteins (Hauser et al. 2015; He et al. 2022), compromise the integrity and sensory function of the lower urinary tract, contributing to clinical manifestations such as pain, hypersensitivity, and voiding dysfunction. Alterations in neural and inflammatory signaling appear to play a central role in this process (He et al. 2022).

In dogs, inflammatory processes of the lower urinary tract may be associated with benign conditions, such as polypoid cystitis and papillomas, as well as malignant neoplasms. Among these, urothelial carcinoma (UC)—also known as transitional cell carcinoma (TCC)—is the most common malignancy in this region, accounting for approximately 2% of all malignant tumors in dogs (Fulkerson and Knapp 2015). Inflammation within the tumor microenvironment plays a critical role in neoplastic progression by stimulating the release of growth factors, pro‐angiogenic mediators, enzymes involved in invasion and metastasis, and immunosuppressive components (Hanahan 2022). In this context, anti‐inflammatory interventions have shown promise, such as piroxicam, a nonsteroidal anti‐inflammatory drug (NSAID) with positive clinical effects in dogs with UC (Knapp et al. 1994; Knapp et al. 2016), although the molecular mechanisms underlying its action remain only partially understood (Knottenbelt et al. 2006).

The enzymes cyclooxygenase‐1 (COX‐1) and cyclooxygenase‐2 (COX‐2) play a central role in mediating inflammation through the oxidative pathway of arachidonic acid, resulting in the formation of prostaglandins—short‐chain lipids involved in both physiological and pathological processes (Souza et al. 2009). COX‐1 is a constitutive enzyme that contributes to tissue homeostasis in various organs, including the stomach, kidneys, intestines, and lungs (Tomlinson and Blikslager 2003). In contrast, COX‐2 is an inducible isoform, typically absent in healthy tissues, but whose expression can be activated by inflammatory cytokines, growth factors, and tumor‐associated stimuli (Spugnini et al. 2005). Prostaglandin E2 (PGE2), the main product of COX‐2, is a potent modulator of inflammation (Jones and Budsberg 2000).

Despite the functional importance of these enzymes, their expression and pathophysiological role in different segments of the lower urinary tract of dogs and cats—such as the bladder and urethra, particularly the proximal urethra, a region richly innervated—remain poorly elucidated (Jung et al. 1999; Shafik, el‐Sibai, and Ahmed 2003; Shafik, Shafik, et al. 2003; Mohammed et al. 2004; Spugnini et al. 2005; Deckmann et al. 2014; Danziger and Grill 2016; Kullmann et al. 2018). Although clinical studies have demonstrated benefits of NSAID use in dogs with UC (Knapp et al. 1994), similar effects have not been consistently observed in cats with FIC (Wallius and Tidholm 2009; Hetrick and Davidow 2013; Macleod et al. 2025), partly due to the lack of well‐designed prospective studies.

Moreover, there are no systematic reviews in the veterinary literature that consolidate current knowledge on COX expression in the lower urinary tract of dogs and cats. This gap limits both the understanding of pathophysiological mechanisms and the development of evidence‐based therapies. The systematization of available information is particularly relevant in light of the growing emphasis on evidence‐based practice in veterinary medicine.

In this context, the aim of this systematic review was to identify, describe, and critically analyze the existing evidence regarding the expression of COX‐1 and COX‐2 in the bladder and urethra of dogs and cats. It also seeks to map knowledge gaps, support future scientific research, and contribute to more rational and species‐specific clinical decision‐making, particularly concerning the use of NSAIDs across different species.

## 2 Materials and Methods

This systematic review was conducted following the PRISMA (Preferred Reporting Items for Systematic Reviews and Meta‐Analyses) guidelines, with the objective of investigating the expression levels of COX‐1 and COX‐2 in bladder and urethral tissues of domestic cats ( *Felis catus* ) and domestic dogs ( *Canis lupus familiaris* ). The database search yielded 669 records. After the removal of 396 duplicates, 273 articles were subjected to an initial screening based on titles and abstracts, independently conducted by two reviewers. In cases of disagreement regarding study eligibility, a third reviewer was consulted to make the final decision.

Original studies specifically addressing the expression of COX‐1 and/or COX‐2 in urethral and/or bladder tissues of cats and dogs, using recognized molecular or immunohistochemical methods, were included. Acceptable methodologies comprised PCR, qPCR, RNA sequencing (RNA‐seq), Western blotting (WB), immunohistochemistry (IHC), immunofluorescence (IF), enzyme‐linked immunosorbent assay (ELISA), Sanger sequencing, proteomics, and next‐generation sequencing (NGS). In addition to original research articles (experimental, observational, or cross‐sectional), systematic reviews and meta‐analyses presenting relevant primary data were also included. Only studies published in English were considered, with no restriction on publication date.

Exclusion criteria encompassed studies that did not include domestic cats or dogs or that employed unrelated animal models; studies that did not specifically investigate COX‐1 or COX‐2; investigations involving tissues other than the urethra or bladder; works that did not present measurable data on enzyme expression; studies that merely mentioned the presence of COX‐1 or COX‐2 without quantifying expression; and articles with inadequate methodology or lacking a description of the quantification methods used.

The literature search was conducted in the PubMed, Scopus, and Web of Science databases, using combinations of controlled descriptors and free‐text terms related to the population, enzymes, and urinary tissues. The terms used included: “cat”, “cats”, “feline”, “kitty”, “ *Felis catus* ”, “domestic cat”, “dog”, “dogs”, “canine”, “ *Canis lupus familiaris* ”, “COX‐1”, “COX‐2”, “cyclooxygenase”, “urethra”, “bladder”, “gene expression”, and “protein expression”, combined with the Boolean operators AND and OR as appropriate. The most recent search update was conducted on April 1, 2025.

**FIGURE 1:**
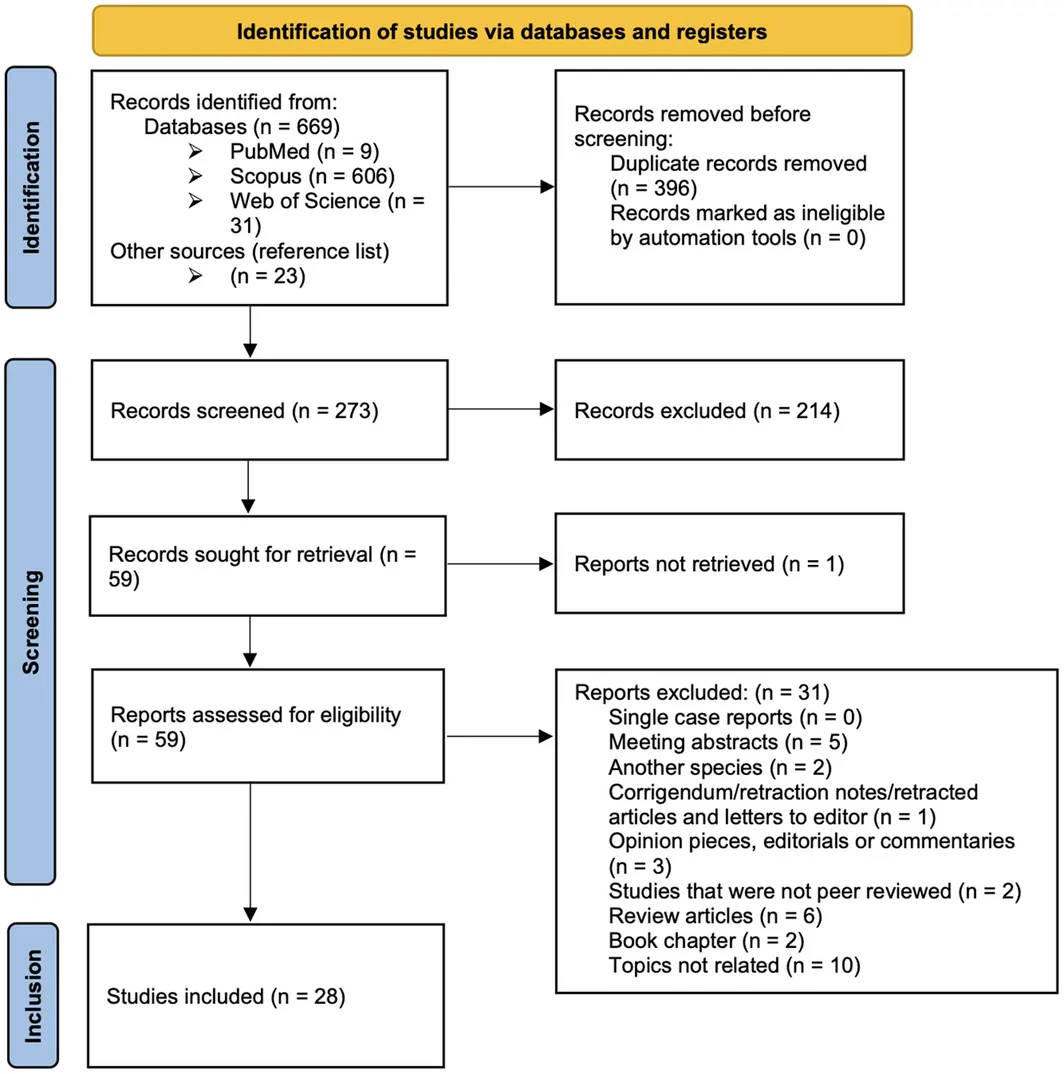
PRISMA flowchart representing the study selection process for the systematic review on the expression of cyclooxygenases in the lower urinary tract of dogs and cats. A total of 669 records were identified through searches in electronic databases (PubMed, Scopus, and Web of Science). After the removal of 396 duplicates, 273 records were screened based on title and abstract. Of these, 214 were excluded for not meeting the eligibility criteria. Fifty‐nine studies were selected for full‐text review, and 28 met the inclusion criteria.

Following the initial screening of titles and abstracts, 59 articles were selected for full‐text review. At this stage, a standardized data extraction table was developed to record the following items for each study: author and year of publication, study location (country and institution), study type (experimental, observational, or review), species and number of animals evaluated (including sex and age data when available), tissue analyzed (urethra, bladder, or both), analytical method employed (such as immunohistochemistry, qPCR, Western blot, among others), data on COX‐1 and COX‐2 expression (presence, absence, intensity, and localization), associated factors (such as inflammation, infection, or neoplasia), main conclusions of the authors, and, when applicable, the level of evidence. The extracted information served as the basis for the final decision regarding study inclusion, according to the predetermined criteria. After data tabulation, 31 studies were excluded, resulting in 28 articles included in this review (Figure 1). This process was independently conducted by two reviewers, with discrepancies resolved by a third reviewer.

## 3 Results

### 3.1 General Characteristics of the Selected Studies

**FIGURE 2:**
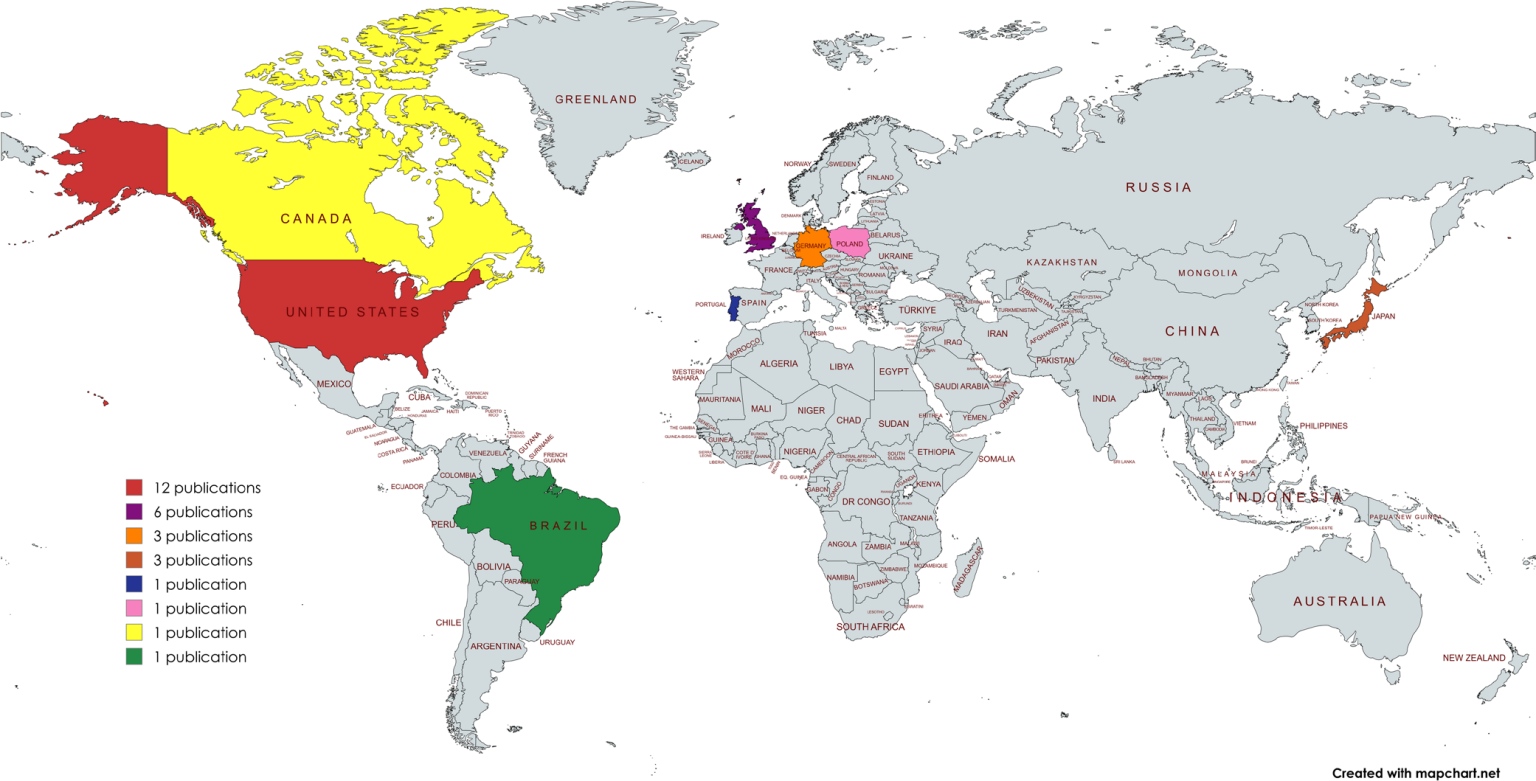
Geographic distribution of the 28 primary studies included in the systematic review on the analysis of COX‐1 and COX‐2 expression in the lower urinary tract of dogs and cats. The colors indicate the number of publications per country, with the United States (*n* = 12), the United Kingdom (*n* = 6), and Japan (*n* = 3) being the most represented, followed by other European and Asian countries with fewer studies.

A total of 28 original articles were included in this review, 15 of which were published between 2000 and 2014, and the remaining 13 within the last 10 years. The selected studies were conducted in various countries, including the United States (*n* = 12), the United Kingdom (*n* = 6), Japan (*n* = 3), Germany (*n* = 3), Portugal (*n* = 1), Poland (*n* = 1), Brazil (*n* = 1), and Canada (*n* = 1) (Figure 2). The review included studies that evaluated the expression of COX‐1 and COX‐2, or COX‐2 alone, in lower urinary tract tissues of dogs and cats. Twelve articles focused specifically on COX‐1 and/or COX‐2 expression in dogs, while three studies evaluated only cats. Some studies also included other species such as humans, horses, and mice, and one study did not use an in vivo model, relying solely on cell lines. The tissues analyzed were primarily the bladder, with the urethra assessed less frequently. Expression was studied in neoplastic tissues—such as TCC of the bladder—as well as in inflamed and healthy tissues. The most commonly used analytical method was IHC, applied alone or in combination with other techniques in nine of the 28 studies. Western blotting was used in five studies. Less frequently employed methods included RNA‐seq bioinformatic analysis, IF, and in situ hybridization (ISH). Reviews addressing COX‐1 and/or COX‐2 expression in domestic animals were also included.

### 3.2 Methods Used for Detecting the Expression of COX‐1 and COX‐2

Various methodological approaches were employed in the studies included in this review with the objective of measuring the expression of the enzymatic isoforms COX‐1 and COX‐2 in tissues such as the bladder and urethra of dogs and cats. IHC stood out as the most widely used technique for detecting protein expression, being present in numerous studies (Khan et al. 2000; Beam et al. 2003; Knottenbelt et al. 2006; Lee et al. 2007; Bommer et al. 2012; Cekanova et al. 2013; Grassinger et al. 2019). IHC allows for the spatial visualization of the target protein in histological sections, enabling the assessment of its cellular distribution and staining intensity—features particularly relevant in neoplastic and inflammatory contexts. In addition to its applicability in formalin‐fixed and paraffin‐embedded tissues, the technique exhibits high specificity when validated antibodies are used and is considered the gold standard for the tissue localization of proteins.

Complementarily, WB was extensively employed, particularly for quantifying COX‐2 expression (Cekanova et al. 2013; Bourn et al. 2019; Yoshitake et al. 2020). WB provides robust quantitative data by enabling electrophoretic separation of proteins according to molecular weight, followed by detection using specific antibodies. In some studies, the technique was used in conjunction with IHC, offering a multidimensional approach; while WB quantifies total protein expression, IHC contextualizes the protein's localization within the tissue, thereby enhancing the robustness of the findings.

Alongside proteomic analyses, investigation of gene expression for PTGS1 (COX‐1) and PTGS2 (COX‐2) was conducted through bioinformatic analyses of RNA‐seq data, as reported in two studies (Yoshitake et al. 2020; Eto et al. 2024). This approach allows for precise quantification of transcripts on a large scale, providing a comprehensive perspective on transcriptional regulation. The integration of transcriptomic and proteomic data enriches the functional interpretation of the COX pathway, particularly in tumorigenic or inflammatory environments.

ISH was employed in the study by Khan et al. (2000) as a strategy to detect PTGS2 transcripts directly in histological sections. By preserving tissue architecture, ISH allows for the spatial localization of mRNA in specific contexts, providing complementary evidence regarding the transcriptional activity of COX‐2 in different cell types.

Other complementary methods were also described. IF, applied in the study by Cekanova et al. (2013), represents an advancement over traditional IHC, offering greater resolution and sensitivity. The use of fluorophore‐conjugated antibodies enables analyses of subcellular co‐localization, as well as the simultaneous assessment of protein expression across multiple markers, which is particularly relevant in characterizing the tumor microenvironment.

Additionally, the study by Packeiser et al. (2020) employed the ELISA to quantify prostaglandin E_2_ (PGE_2_), one of the primary metabolites derived from the enzymatic activity of COX‐2. Although ELISA does not directly assess enzyme expression, the measurement of its products provides a functional inference regarding its activity, representing an important tool in evaluating the biological impact of the COX pathway.

It is noteworthy that certain studies addressed the presence or role of COXs in a descriptive or conceptual manner, without detailing the methodologies used for quantification (Mohammed et al. 2004; De Nardi et al. 2011; Doré 2011; Erol et al. 2012; Charlton et al. 2013; Dorsch et al. 2016; Ito et al. 2018; Bourn et al. 2019; Clerc‐Renaud et al. 2021; Gregório et al. 2021; Cerdà et al. 2023). Other works, such as by Flory and LeBlanc (2005), discussed the involvement of these enzymes in pathological processes but did not report the analytical techniques employed for their detection.

The methodologies applied in each study are systematically organized in Table 1, allowing for a comparative analysis of the different approaches and their frequency of use in the reviewed literature.

**TABLE 1:** Methods used for assessing the expression of COX‐1 and/or COX‐2 in the studies included in this systematic review.

Study title	Species	Tissue/Type of neoplasm	COX evaluated	Detection methods	References
Characterization of six canine prostate adenocarcinoma and three transitional cell carcinoma cell lines derived from primary tumour tissues as well as metastasis	Canine	Tumour cells (cell lines)	COX‐2	IHC; ELISA for PGE2	Packeiser et al. (2020)
Aberrant expression of the COX2/PGE2 axis is induced by activation of the RAF/MEK/ERK pathway in BRAFV595E canine urothelial carcinoma	Canine	Urothelial (UC with BRAF mutation)	COX‐1, COX‐2	IHC; WB; RNA‐seq	Yoshitake et al. (2020)
An immunohistochemical study of cyclooxygenase‐2 expression in various feline neoplasms	Feline	Diverse neoplasms	COX‐2	IHC	Beam et al. (2003)
Correlation of BRAF Variant V595E, breed, histological grade and bladder cyclooxygenase‐2 expression in canine transitional cell carcinomas	Canine	Bladder TCC	COX‐2	IHC	Grassinger et al. (2019)
Clinical features, survival times and COX‐1 and COX‐2 expression in cats with transitional cell carcinoma of the urinary bladder treated with meloxicam	Feline	Bladder TCC	COX‐1, COX‐2	IHC	Bommer et al. (2012)
Cohort study of COX‐1 and COX‐2 expression in canine rectal and bladder tumours	Canine	Bladder TCC and rectal tumours	COX‐1, COX‐2	IHC	Knottenbelt et al. (2006)
Comprehensive analysis of the tumour immune microenvironment in canine urothelial carcinoma reveals immunosuppressive mechanisms induced by the COX‐prostanoid cascade	Canine	UC	COX‐1, COX‐2	RNA‐seq	Eto et al. (2024)
Detection of tyrosine kinase inhibitors‐induced COX‐2 expression in bladder cancer by fluorocoxib A	Canine	Bladder TCC	COX‐2	WB	Bourn et al. (2019)
Expression of cyclooxygenase‐2 in transitional cell carcinoma of the urinary bladder in dogs	Canine	Bladder TCC	COX‐2	IHC; ISH	Khan et al. (2000)
Molecular imaging of cyclooxygenase‐2 expression in canine transitional cell carcinomas in vitro and in vivo	Canine	Bladder TCC	COX‐2	IHC; WB; IF	Cekanova et al. (2013)
Expression of cyclooxygenase‐2, P‐glycoprotein and multi‐drug resistance‐associated protein in canine transitional cell carcinoma	Canine	Bladder TCC	COX‐2	IHC	Lee et al. (2007)
Expression of cyclooxygenase‐2 in the canine lower urinary tract with regard to the effects of gonadal status and gender	Canine	Lower urinary tract (bladder and urethra), non‐neoplastic	COX‐2	IHC; ISH	Ponglowhapan et al. (2009)
Differences in expression of uroplakin III, cytokeratin 7, and cyclooxygenase‐2 in canine proliferative urothelial lesions of the urinary bladder	Canine	Proliferative urothelial lesions (bladder)	COX‐2	IHC	Sledge et al. (2015)
Inflammation and tissue remodeling in the bladder and urethra in feline interstitial cystitis	Feline	Bladder and urethra (FIC)	COX‐1, COX‐2	IHC	Kullmann et al. (2018)
Lipoxygenase‐5 expression in canine urinary bladder: normal urothelium, cystitis and transitional cell carcinoma	Canine	Bladder: normal, cystitis, TCC	COX‐1, COX‐2	IHC	Finotello et al. (2019)
Review or clinical studies without direct COX measurement	Canine/Feline	Diverse	—	Not applicable or method not described	Flory and LeBlanc (2005), Hayes (2007), De Nardi et al. (2011), Doré (2011), Erol et al. (2012), Rahnama'i et al. (2012), Charlton et al. (2013), Dorsch et al. (2016), Bourn and Cekanova (2018), Ito et al. (2018), Clerc‐Renaud et al. (2021), Gregório et al. (2021), Cerdà et al. (2023)

*Note:* This table summarises the selected studies and the methods used to assess the expression of cyclooxygenase isoforms COX‐1 and/or COX‐2 in urinary tract tissues of dogs and cats. The approaches include protein‐based techniques, such as immunohistochemistry (IHC), Western blotting (WB), immunofluorescence (IF), and ELISA, as well as molecular methods, including in situ hybridisation (ISH) and RNA‐seq‐based bioinformatic analysis.

Abbreviations: FIC, feline idiopathic/interstitial cystitis; TCC, transitional cell carcinoma; UC, urothelial carcinoma.

### 3.3 Expression of COX‐1 and COX‐2 in the Bladder and Urethra of Felines

The characterization of cyclooxygenase isoform expression (COX‐1 and COX‐2) in the lower urinary tract of felines has revealed distinct patterns between urethral and vesical compartments, with relevant pathophysiological and therapeutic implications, although the available data remain limited and heterogeneous.

In normal bladder tissues of cats, COX‐2 is not detected, suggesting a lack of constitutive expression of this isoenzyme in the urothelium under physiological conditions (Bommer et al. 2012). In contrast, COX‐1 is expressed in the bladder mucosa of healthy animals, as demonstrated in studies comparing normal and pathological tissues, such as in FIC and TCC (Kullmann et al. 2018). However, the isolated statistical significance of COX‐1 expression in normal bladder tissues was not emphasized, as the analyses were primarily comparative in nature.

In FIC, a significant increase in COX‐1 expression was observed in the bladder mucosa of affected cats, with no significant change in COX‐2 expression in the bladder (Kullmann et al. 2018). Nevertheless, a marked elevation in the production of inflammatory prostanoids, such as PGE_2_, was noted, reinforcing the activation of the inflammatory pathway and supporting the investigation of COX‐2 inhibitors as a therapeutic approach (Kullmann et al. 2018). Indeed, meloxicam, an NSAID with preferential COX‐2 selectivity has shown paliative benefit in cats with bladder TCC, with clinical improvement (reduction of hematuria and/or dysuria) in most treated cats (Bommer et al. 2012). In obstructive FIC, the increased urethral COX‐2 expression provides a rational for COX‐2 inhibition (Kullmann et al. 2018); however, the only randomized controlled trial to date found that meloxicam did not reduce the rate of recurrent urethral obstruction or hasten recovery (Dorsch et al. 2016).

COX‐2 expression in neoplastic feline bladder tissue has been reported at variable frequencies. Beam et al. (2003) identified positivity in 37% of the evaluated cases (7/19), while Bommer et al. (2012), in a smaller sample (*n* = 7), reported COX‐2 positivity in 71% of tumors, generally characterized by focal staining (in fewer than 1% of cells), but with moderate to strong cytoplasmic intensity. COX‐1, in contrast, was universally expressed in all TCC cases analyzed in these studies.

The prognostic implication of COX‐2 expression in feline bladder tumors was suggested by (Bommer et al. 2012), who reported a shorter median survival in cats with COX‐2–positive tumors (123 days) compared to those with COX‐2–negative tumors (375 days). However, this difference did not reach statistical significance, possibly due to the small sample size, the retrospective nature of the study, treatment heterogeneity, and the lack of a standardized cutoff point for defining positivity.

While data regarding the bladder are relatively more consistent, COX expression in feline urethral structures remains largely unexplored. Only one study was identified through a rigorous literature search that assessed COX‐1 and COX‐2 expression in the urethra of cats with FIC (Kullmann et al. 2018). In that analysis, conducted on the proximal urethra, no significant difference in constitutive COX‐1 expression was found between control (*n* = 12) and FIC (*n* = 14) groups. However, COX‐2 expression was significantly increased in the urethral mucosa, specifically in the lamina propria, of cats with FIC compared to controls.

In addition to the increased COX‐2 expression in the proximal urethra, relevant histological alterations were observed in the same region, including leukocyte accumulation (in 3 out of 4 cats), presence of von Brunn's nests (in 3 out of 7 cats), pronounced but not statistically significant angiogenesis, and a significant increase in elastin deposition (in 7 cats with FIC versus 3 in the control group) (Kullmann et al. 2018). These findings support the hypothesis of a localized inflammatory process in the urethra, with structural and cellular features that may contribute to the pathophysiology of FIC.

In summary, the currently available data indicate that, in cats, COX‐1 is constitutively expressed in the bladder and remains stable in the urethra even under inflammatory conditions, whereas COX‐2 expression is induced in response to inflammation, with a distinct pattern between the bladder and urethra. The increase in COX‐2 expression in the urethra—but not in the bladder—in FIC, along with its association with inflammatory tissue alterations, suggests a specific pathogenic role for this isoenzyme in the proximal urethra. In bladder neoplasms, COX‐2 is present in a subset of tumors and may have prognostic value, although this remains inconclusive. These findings underscore the importance of COX‐2 as a therapeutic target and justify further investigations to clarify its role in the pathophysiology and clinical management of urinary tract disorders in felines.

### 3.4 Expression of COX‐1 and COX‐2 in the Bladder and Urethra of Canines

The expression of COX‐1 and COX‐2 in the canine lower urinary tract has been investigated in various physiological and pathological contexts, revealing distinct patterns between the urethra and bladder, as well as the influence of hormonal and sex‐related factors.

In the urethral tissue of healthy dogs, Ponglowhapan et al. (2009) demonstrated that COX‐2 and its mRNA are constitutively expressed in both the proximal and distal urethra. This expression varied according to anatomical location (bladder body and neck, proximal and distal urethra) and the histological layers evaluated (epithelium, subepithelial stroma, and smooth muscle). Significant differences were observed between sexes and between intact and gonadectomized animals. Intact females exhibited higher overall COX‐2 expression, particularly in epithelial layers, except in the proximal urethra, where no sex‐based differences were observed. In contrast, among neutered dogs, males showed higher enzyme expression across all regions and layers analyzed. The reduced COX‐2 expression in gonadectomized dogs—especially females—was associated with a possible impairment of lower urinary tract functional homeostasis and identified as a potential predisposing factor for post‐spaying urinary incontinence (Ponglowhapan et al. 2009).

In contrast, most studies on COX enzymes in dogs with UC, formerly referred to as TCC, have focused on the urinary bladder. In normal bladder tissues, COX‐1 has been consistently detected in the epithelium, whereas COX‐2 is either absent or present at negligible levels, reinforcing its non‐constitutive nature and its induction under pathological conditions (Khan et al. 2000; Finotello et al. 2019). In bladder neoplasms, COX‐2 expression was markedly increased in both primary and metastatic tumor cells, with positivity ranging from 58% to 100% (Lee et al. 2007; Khan et al. 2000) and 100% in another that analyzed 21 cases (Khan et al. 2000). Expression intensity varied from absent to strong, with no significant correlation with tumor histological grade (Grassinger et al. 2019).

In addition, COX‐1 expression was also identified in tumors, although to a lesser extent. An upregulation of mPGES‐1 and mPGES‐2 was observed in neoplastic tissues, indicating activation of the prostanoid pathway associated with tumor progression (Eto et al. 2024). Studies have also identified an association between BRAF mutations and higher COX‐2 expression in non‐terrier breeds, suggesting a more aggressive tumor phenotype with heightened activation of this pathway (Grassinger et al. 2019).

From a therapeutic perspective, COX‐2 inhibitors, such as piroxicam, have demonstrated clinical efficacy in controlling canine UC, despite intratumoral levels of COX‐2 or PGE_2_ not being reliable predictors of response or prognosis (Khan et al. 2000; Doré 2011). Inhibition of the COX‐2/mPGES‐2 pathway has also been associated with reversal of tumor‐induced immunosuppression: dogs treated with COX inhibitors showed increased infiltration of CD3^+^ and CD8^+^ T cells within tumor nests, suggesting the conversion of a “cold tumor” into a “hot tumor” (Eto et al. 2024). In oncologic immunology, cold tumors are defined by scarce immune‐cell infiltration, low cytotoxic T‐cell engagement and a predominantly immunosuppressive microenvironment, typically resulting in poor responsiveness to immunomodulatory therapies. Conversely, hot tumors display dense infiltration of effector lymphocytes—particularly CD8^+^ T cells—together with a pro‐inflammatory microenvironment and increased immunogenicity, features associated with more effective antitumor immune responses (Galon and Bruni 2019). Moreover, molecular imaging technologies such as fluorocoxib A have demonstrated the ability to selectively detect COX‐2–expressing bladder tumors in vivo during cystoscopy, without labeling normal urothelium; uptake was significantly reduced following COX‐2 blockade with celecoxib (Cekanova et al. 2013).

Collectively, these findings indicate that COX‐1 is constitutively expressed in the normal canine bladder epithelium, whereas COX‐2 is absent under physiological conditions but widely expressed in urothelial tumors. In the urethra, COX‐2 is expressed even in normal tissues, with evident hormonal and sex‐based modulation, suggesting a relevant physiological role. The marked overexpression of COX‐2 in neoplasms, its association with specific mutations, tumor immunosuppression, and response to NSAIDs support its value as both a diagnostic and therapeutic target in canine urothelial carcinoma.

### 3.5 Comparison Between Species

Comparative analyses between dogs and cats reveal both similarities and differences in the expression and clinical implications of COX enzymes. In both species, COX‐1 is constitutively expressed in normal bladder tissues, whereas COX‐2 is absent under physiological conditions and primarily detected in pathological states such as inflammation and neoplasia (Khan et al. 2000; Bommer et al. 2012; Finotello et al. 2019).

However, felines exhibit a distinct pattern in feline idiopathic cystitis (FIC), characterized by increased COX‐1 expression in the bladder and elevated COX‐2 expression in the urethra (Kullmann et al. 2018). In contrast, in dogs, COX‐2 is constitutively expressed in the lower urinary tract, particularly in the proximal urethra, with modulation by sex and gonadectomy status (Ponglowhapan et al. 2009). This basal COX‐2 expression in canines suggests additional physiological roles for the enzyme in maintaining lower urinary tract homeostasis.

Regarding bladder cancer, both species demonstrate overexpression of COX‐2 in tumors; however, the frequency and intensity of expression appear to be higher in dogs. Therapeutic response to COX‐2 inhibition is also better documented in dogs, with evidence of clinical and immunological benefits (Doré 2011; Eto et al. 2024). In cats, although there are indications that COX‐2 positivity may be associated with poorer prognosis (Gregório et al. 2021), the limited number of studies precludes definitive conclusions.

These differences highlight species‐specific pathophysiological characteristics that should be taken into account when selecting diagnostic and therapeutic approaches for lower urinary tract diseases.

## 4 Discussion

The analysis of available data reveals distinct expression patterns of cyclooxygenase isoforms (COX‐1 and COX‐2) in the LUT of dogs and cats, which have important pathophysiological, clinical, and prognostic implications. In felines, COX‐1 is constitutively expressed in the bladder mucosa under physiological conditions, whereas COX‐2 is not detected in normal bladder tissues (Bommer et al. 2012; Kullmann et al. 2018). In the context of FIC, a significant increase in COX‐1 expression is observed in the bladder, without changes in COX‐2 expression in this tissue. Conversely, the urethral mucosa of cats with FIC, particularly the lamina propria of the proximal urethra, exhibits a marked increase in COX‐2 (Kullmann et al. 2018), suggesting a localized activation of the inflammatory pathway in this segment.

In dogs, COX‐1 expression is also constitutive in the normal bladder epithelium (Khan et al. 2000), whereas COX‐2 is virtually absent in healthy tissues but is widely expressed in UC (De Nardi et al. 2011; Gregório et al. 2021). It is noteworthy that COX‐2 can be physiologically expressed in the canine urethra, with variations influenced by sex and hormonal status, being more intensely expressed in intact animals, particularly females (Ponglowhapan et al. 2009).

These expression patterns suggest clinically relevant implications. In cats with FIC, the increased expression of COX‐2 in the urethra, but not in the bladder, supports the hypothesis that the inflammatory process is primarily localized in the urethral segment. This finding provides a rational for the use of selective COX‐2 inhibitors, such as meloxicam, which have demonstrated efficacy in improving urinary parameters in cases of obstructive FIC (Kullmann et al. 2018); to date, however, a randomized controlled trial did not demonstrate a clinical benefit of meloxicam on recurrent obstruction or clinical recovery (Dorsch et al. 2016). Although bladder COX‐2 expression remains unchanged, the elevation of inflammatory prostanoids such as PGE_2_ indicates activation of the inflammatory pathway, further corroborating the therapeutic approach with COX‐2 inhibitors (Kullmann et al. 2018).

In dogs, the physiological expression of COX‐2 in the lower urinary tract, modulated by sex and hormonal factors, has specific clinical implications. The reduction of this expression following gonadectomy may compromise the functional integrity of the urethra, particularly in females, which could be associated with the higher prevalence of urinary incontinence observed post‐sterilization in this population (Ponglowhapan et al. 2009).

In the neoplastic scenario, both in dogs and cats, COX‐2 shows elevated expression in bladder tumors, although with variable intensities and frequencies between species. In felines, COX‐2 was detected in 37% to 71% of TCC (Beam et al. 2003; Bommer et al. 2012), whereas in dogs, its presence was observed in 88% to 100% of the cases evaluated (Cekanova et al. 2013; Gregório et al. 2021). COX‐1, on the other hand, presents consistent expression in both normal and neoplastic tissues in both species (Khan et al. 2000; Bommer et al. 2012).

The high expression of COX‐2 in tumors suggests its involvement in carcinogenesis and modulation of the tumor microenvironment. In dogs, inhibition of this enzyme by NSAIDs, such as piroxicam, has demonstrated therapeutic efficacy, although intratumoral COX‐2 levels are not reliable predictors of treatment response (Khan et al. 2000; Doré 2011). Additionally, there is evidence that COX‐2 promotes tumor immunosuppression, and its blockade can increase infiltration of CD3^+^ and CD8^+^ T lymphocytes, fostering a more robust antitumor immune response (Eto et al. 2024). Advances in identifying COX‐2‐positive tumors include the use of molecular imaging techniques, such as the application of fluorocoxib A during cystoscopy, which allows specific and targeted visualization of lesions (Cekanova et al. 2013).

In cats, although the prognostic role of COX‐2 still requires clarification, there is evidence that its expression is associated with shorter survival in positive cases (Gregório et al. 2021), suggesting a potential prognostic value to be further explored.

Despite these promising findings, this review identified important limitations in the available studies. Among them are the small sample sizes, lack of methodological standardization, and absence of control for variables such as comorbidities and clinical stage of the disease. The heterogeneity of techniques used for biomarker detection, diagnostic criteria adopted, and the way data are presented make direct comparisons between studies difficult.

Considering these limitations, future research should prioritize prospective study designs with larger samples and standardized inclusion criteria. The validation of the identified biomarkers requires the use of robust statistical analyses, such as sensitivity, specificity, and ROC curves, as well as correlation of findings with disease stage and clinical outcomes. Multicenter, collaborative studies with a multiparametric approach have great potential to increase reproducibility, diagnostic accuracy, and clinical applicability of biomarkers associated with COX pathways in the lower urinary tract of dogs and cats.

## 5 Conclusion

The analysis of COX‐1 and COX‐2 expression in the lower urinary tract of dogs and cats revealed significant differences between species, tissues, and clinical conditions, with relevant implications for veterinary medicine, particularly in felines. COX‐1 exhibited a constitutive expression pattern in the bladder of both species, whereas COX‐2 was absent in normal tissues except in the canine urethra, suggesting a local physiological role. In cats with FIC, increased COX‐1 expression was observed in the bladder alongside a significant elevation of COX‐2 in the proximal urethra, indicating a possible involvement in the perpetuation of urethral inflammation. In UC, COX‐2 expression was more frequent in dogs but also present in felines, with potential prognostic associations. These findings reinforce the role of COX enzymes, particularly COX‐2, in the pathophysiology of inflammatory and neoplastic diseases of the lower urinary tract, and provide a rational for further investigation of selective COX‐2 inhibitors as an adjuvant therapeutic alternative, especially in cats with FIC. The scarcity of studies focusing on the feline and canine urethra, in both inflammatory and neoplastic contexts, underscores the need for further investigations with larger case series and methodological standardization, aimed at deepening the understanding of the role of these enzymes and their clinical applicability.

## Author Contributions

All authors were involved in conception and design of this manuscript. R.S.M. and L.G.D.B. conducted the investigation. R.S.M., L.G.D.B., A.G.G.R.C., J.F.F., S.R.M., D.R., D.G.S.R., A.M. and P.T.C.G.‐O. analyzed and interpreted the data. R.S.M. and L.G.D.B. prepared the original draft. All authors were involved in review and editing.

## Funding

The authors have nothing to report.

## Ethics Statement

The authors have nothing to report.

## Conflicts of Interest

The authors declare no conflicts of interest.

## Data Availability

Data sharing not applicable to this article as no datasets were generated or analysed during the current study.
